# Visual Outcomes After Mix-and-Match Implantation of Trifocal and Extended Depth-of-Focus Intraocular Lenses: A Systematic Review and Meta-Analysis

**DOI:** 10.3390/medicina62061112

**Published:** 2026-06-08

**Authors:** Meruyert Rakhimova, Neilya Aldasheva, Ardak Auyezova, Lukpan Orazbekov, Zauresh Utelbayeva, Shnara Svetlanova, Indira Karibayeva

**Affiliations:** 1 Kazakhstan’s Medical University “KSPH”, Almaty 050060, Kazakhstan; rakhimovameruyert06@gmail.com (M.R.); auezova_ardak@mail.ru (A.A.); 2 Department of Science Management, Kazakh Eye Research Institute, Almaty 050012, Kazakhstan; n.aldasheva@eyeinst.kz (N.A.); lukpan.orazbekov@gmail.com (L.O.); 3 Department of Ophthalmology, S.D. Asfendiyarov Kazakh National Medical University, Almaty 050012, Kazakhstan; 4 Department of Nursing, Kazakhstan Medical University “KSPH”, Almaty 050060, Kazakhstan; svetlanova.sh@kaznmu.kz; 5 Department of Research Management, JSC Research Institute of Cardiology and Internal Diseases, Almaty 050012, Kazakhstan; ik01379@georgiasouthern.edu; 6 Jiann-Ping Hsu College of Public Health, Georgia Southern University, Statesboro, GA 30460, USA

**Keywords:** cataract surgery, presbyopia-correcting intraocular lenses, trifocal intraocular lens, extended depth of focus, mix-and-match implantation, binocular visual acuity, postoperative visual outcomes

## Abstract

*Background and Objectives*: The use of presbyopia-correcting intraocular lenses (IOLs) has become an integral part of modern cataract surgery. Trifocal IOLs are designed to provide functional vision at multiple distances but may be associated with dysphotopsia and reduced contrast sensitivity. In contrast, extended depth-of-focus (EDOF) IOLs offer a smoother defocus profile and fewer photic phenomena; however, they often provide insufficient uncorrected near visual acuity. To overcome the limitations of each design, a mix-and-match implantation strategy, involving implantation of a trifocal IOL in one eye and an EDOF IOL in the fellow eye, has been proposed. The objective of this systematic review was to evaluate whether mix-and-match implantation can maintain high visual acuity at near, intermediate, and distance ranges in the early postoperative period. *Materials and Methods*: A systematic literature search was conducted in five electronic databases, including PubMed, Scopus, Web of Science, Google Scholar, and ScienceDirect, following PRISMA guidelines. Studies reporting outcomes of mix-and-match implantation with a trifocal IOL in one eye and an EDOF IOL in the fellow eye were included. Only studies presenting binocular uncorrected visual acuity (UCVA) at a distance, intermediate, and near ranges, expressed in logMAR units, were considered. Visual outcomes assessed at approximately 3 months postoperatively were extracted. *Results*: Five studies involving 225 patients were included. The pooled mean logMAR UDVA at 3 months was 0.05 (95% CI: 0.03–0.07), with substantial heterogeneity (I^2^ = 80.1%). The pooled mean logMAR UNVA was 0.09 (95% CI: 0.05–0.14), with high heterogeneity (I^2^ = 87.9%). For intermediate vision, the pooled mean logMAR UIVA was 0.03 (95% CI: −0.00 to 0.06), with moderate heterogeneity (I^2^ = 72.5%). Meta-regression analyses did not show statistically significant associations between publication year and visual outcomes for UDVA (*p* = 0.695), UNVA (*p* = 0.469), or UIVA (*p* = 0.099). Sensitivity analyses confirmed the robustness of the pooled estimates. *Conclusions*: Mix-and-match implantation of a trifocal IOL in one eye and an EDOF IOL in the fellow eye provides favorable early binocular visual acuity across distance, intermediate, and near ranges. However, substantial heterogeneity across studies and very low certainty of evidence warrant cautious interpretation of these findings.

## 1. Introduction

Cataract surgery has undergone a profound transformation over the past few decades. What was once a procedure aimed primarily at restoring transparency to an opacified lens has evolved into one of the most precise refractive surgeries in modern medicine [1].

Cataracts remain the leading cause of reversible visual impairment worldwide. According to the Global Burden of Disease 2021 data, the global burden rate of cataracts in 2021 was 1181 cases per 100,000 population and is continuing to rise [2]. Therefore, phacoemulsification with intraocular lens (IOL) implantation is one of the most frequently performed surgical procedures globally [3]. Modern ophthalmic surgery has evolved beyond simple cataract extraction into a highly precise refractive discipline, where the creation of an individualized visual profile has become a primary objective [1,4,5]. This shift has been facilitated by the development of premium intraocular lenses (IOLs), among which multifocal and extended depth of focus (EDOF) designs play a central role [6,7]. However, each optical design inherently represents a trade-off between range of vision and visual quality [8,9].

Trifocal intraocular lenses are based on optical designs that provide functional vision at distance, intermediate, and near ranges. This simultaneous-vision design enables high levels of spectacle independence and provides good uncorrected near and distance visual acuity, making these lenses particularly attractive for patients prioritizing reading and close work [10]. However, because light energy is distributed among multiple foci, contrast sensitivity may be reduced and photic phenomena such as halos and glare can occur, especially under mesopic conditions. Thus, while trifocal IOLs offer strong near performance and a broad functional range, their optical mechanism inherently involves trade-offs in visual quality [11].

EDOF lenses aim to create an elongated focal area and provide a smoother transition between distance and intermediate vision [12,13]. This optical approach tends to provide smoother intermediate vision and a more favorable dysphotopsia profile compared with traditional multifocal designs. Nevertheless, because EDOF lenses do not provide a distinct high-add near focus, near visual acuity may be less pronounced than that achieved with trifocal IOLs, particularly for fine print [13].

In clinical practice, the challenge lies in balancing near performance, intermediate functionality, contrast sensitivity, and the risk of dysphotopsia while maintaining overall patient satisfaction [14,15]. In summary, trifocal IOLs typically provide good near visual acuity, yet this benefit may come at the cost of increased halos and reduced contrast sensitivity compared with monofocal lenses [16]. Conversely, EDOF lenses are often associated with smoother intermediate performance and fewer dysphotopsias, although near visual acuity may be less pronounced than that achieved with trifocal designs [15]. Thus, surgeons frequently face a clinical dilemma: prioritizing one visual domain may compromise another.

The absence of a universal solution has led to the emergence of mix-and-match implantation strategies and commercially developed binocular systems, aiming to overcome the limitations of individual lenses through the synergistic combination of two different IOL models [4,17]. By combining complementary optical profiles, such as a trifocal lens for enhanced near performance in one eye and an EDOF lens for improved intermediate function and visual quality in the fellow eye, it may be possible to leverage binocular summation and neural adaptation to achieve a broader and more balanced functional range of vision. Unlike standardized bilateral protocols, this technique allows the surgeon to independently select each IOL based on individual patient characteristics and visual priorities. Such flexibility enables a more personalized refractive plan, adapting lens selection to specific lifestyle demands and visual expectations [4].

However, despite its theoretical rationale and increasing clinical adoption, the evidence supporting trifocal–EDOF pairing remains fragmented. Published studies vary considerably in study design, patient selection criteria, IOL combinations, outcome measures, and follow-up intervals [18,19]. Reported endpoints range from monocular and binocular visual acuity to defocus curves, contrast sensitivity, dysphotopsia assessment, and patient-reported outcomes, often measured under different testing conditions [16,19,20]. Such methodological heterogeneity complicates cross-study comparison and limits the ability to determine whether the perceived advantages of mix-and-match implantation are consistent and clinically meaningful.

Moreover, many available investigations are non-randomized, involve relatively small cohorts, and differ in the specific optical platforms combined, further contributing to variability in reported outcomes [19]. While some studies suggest improved intermediate performance or enhanced patient satisfaction, others report comparable results for bilateral implantation strategies, making it difficult to define the true benefit–risk profile of asymmetric lens pairing [21,22].

Only a few studies have specifically evaluated binocular outcomes at the same postoperative timepoints in patients who received a trifocal IOL in one eye and an EDOF IOL in the fellow eye. However, binocular vision is what ultimately determines functional performance after presbyopia-correcting surgery. Monocular results alone do not fully reflect how patients see in everyday life. Therefore, available evidence needs to be summarized in a structured way to understand whether the theoretical optical complementarity of these lenses translates into a real functional benefit. This issue is clinically important. Modern cataract and refractive lens exchange surgery is increasingly individualized, and patients expect reliable vision at distance, intermediate, and near ranges. In the absence of clear data, surgeons often rely on indirect comparisons or personal experience when recommending a mix-and-match strategy.

For this reason, a systematic and quantitative evaluation of existing studies is necessary to better define binocular visual outcomes and support patient selection. The aim of this systematic review and meta-analysis is to synthesize available evidence on binocular uncorrected visual acuity at distance, intermediate, and near three months after implantation of a trifocal IOL in one eye and an EDOF IOL in the fellow eye.

## 2. Materials and Methods

### 2.1. Study Registration

A systematic review was conducted in accordance with the Preferred Reporting Items for Systematic Reviews and Meta-Analyses 2020 (PRISMA 2020) guidelines [23]. The completed PRISMA 2020 checklist is provided in Supplementary Table S1. The review was designed to evaluate visual outcomes following mix-and-match implantation strategies involving trifocal and extended depth-of-focus (EDOF) intraocular lenses in patients undergoing cataract surgery. The review protocol was registered with the International Prospective Register of Systematic Reviews (PROSPERO) (ID: CRD420251252326, date: 24 December 2025).

### 2.2. Search Strategy

A comprehensive literature search was performed to identify relevant studies published up to the end of 2025. The following electronic databases were systematically searched: PubMed, Scopus, Web of Science, Google Scholar, and ScienceDirect. No restrictions were applied regarding the year of publication.

Search terms were developed based on preliminary screening of titles and abstracts of publications related to mixed intraocular lens implantation strategies. The search strategy combined terminology related to mix-and-match implantation approaches and presbyopia-correcting IOL designs. The search strategy included combinations of “mix and match,” “mix-and-match,” “mix & match,” “blended vision,” “trifocal,” “trifocal IOL,” “extended depth of focus,” “EDOF,” “intraocular lens,” and “IOL.” As there is no single MeSH term that captures this concept, free-text keywords were necessary to ensure comprehensive coverage. The search strategy was adapted for each database as appropriate. Further details of the search strategy are provided in Supplemental Table S2.

### 2.3. Eligibility Criteria, Study Selection and Data Collection

Supplemental Table S3 summarizes the eligibility criteria applied for study selection using the Population, Intervention, Comparator, Outcome, and Study Design (PICOS) framework.

The population consisted of adult patients undergoing cataract surgery. Studies that included patients with congenital, traumatic, or complicated cataracts; a pediatric population; patients with ocular comorbidities potentially affecting postoperative visual outcomes (advanced glaucoma, age-related macular degeneration, or diabetic retinopathy); or a history of previous refractive or intraocular surgery were excluded. Studies involving unilateral implantation, bilateral implantation of the same IOL type, or monofocal intraocular lenses only were also excluded.

The intervention of interest was a mixed intraocular lens implantation strategy, involving the combined implantation of trifocal intraocular lenses and extended depth-of-focus (EDOF) intraocular lenses, also referred to as a mix-and-match approach.

No comparator was defined.

The outcomes of interest included uncorrected visual acuity at distance (UDVA), intermediate (UIVA), and near ranges (UNVA), reported in logMAR units, with assessments conducted at a postoperative follow-up of 3 months. Studies that failed to report quantitative visual acuity outcomes or used non-standardized measurement units were excluded. Eligible study designs included prospective and retrospective observational studies, case series, registry-based studies, and non-randomized comparative studies. Randomized controlled trials were excluded because the available evidence base was predominantly observational and highly heterogeneous in design, patient selection, IOL combinations, and outcome reporting. Restricting the quantitative synthesis to observational and non-randomized studies was intended to improve methodological comparability across the included studies and avoid combining fundamentally different study designs within a small evidence pool. Reviews, meta-analyses, conference abstracts, editorials, letters, and commentaries were excluded. Only studies published in English-language peer-reviewed journals were considered.

Two reviewers independently conducted the literature search and screening process (M.R. and I.K.). Following database searches, all retrieved records were collated in a single database, and duplicate entries were identified and removed using Mendeley Reference Manager, version 2.142.0 (Elsevier B.V., Amsterdam, The Netherlands). Titles and abstracts of unique records were initially screened for relevance. Subsequently, full-text articles were assessed for eligibility based on the predefined inclusion criteria. Data extraction was carried out using a standardized extraction form developed for this review.

The extracted data included the first author’s name, year of publication, country of origin, study design, sample size, patient demographics, type and combination of implanted intraocular lenses, duration of postoperative follow-up, and uncorrected visual acuity outcomes at distance, intermediate, and near reported in logMAR units.

When available, data on ocular dominance, allocation of the trifocal or EDOF IOL to the dominant or non-dominant eye, and refractive targeting strategy, including micromonovision, were also extracted. However, these variables were not consistently reported across all included studies.

The extracted datasets from both reviewers were compared and merged. Any disagreements regarding study inclusion or data extraction were resolved through discussion with the third author (Z.U.) until consensus was achieved.

### 2.4. Meta-Analysis

All quantitative syntheses were conducted in Rversion 4.5.2 (R Foundation for Statistical Computing, Vienna, Austria) using RStudio (Posit Software, PBC, Boston, MA, USA), version 2023.12.1 + 402 [24,25]. Meta-analyses were performed for postoperative visual acuity outcomes at 3 months and reported in logMAR units. A random-effects model was prespecified for all pooled analyses to account for expected between-study variability in study design, patient characteristics, intraocular lens combinations, and clinical settings. Pooled mean estimates with corresponding 95% confidence intervals (CIs) were calculated for each outcome. Between-study variance was estimated using the restricted maximum likelihood (REML) method.

Statistical heterogeneity was assessed using Cochran’s Q test, the I^2^ statistic, and τ^2^ (tau-squared). Heterogeneity was interpreted using conventional thresholds for I^2^ (approximately 25% = low, 50% = moderate, and 75% = high heterogeneity), while also considering the magnitude of τ^2^ and the clinical/methodological diversity of the included studies. Because heterogeneity was anticipated, additional exploratory analyses were planned a priori. To investigate potential sources of heterogeneity, meta-regression analyses were conducted using random-effects REML models with year of publication and mean participant age as study-level moderators. Because of the limited number of included studies, all meta-regression analyses were considered exploratory and hypothesis-generating only. The sensitivity of the pooled estimates was further examined using leave-one-out analyses, in which each study was sequentially omitted to evaluate its influence on the pooled effect estimate and heterogeneity.

Assessment of publication bias was planned only when at least 10 studies were available for a given outcome, in line with methodological recommendations for meta-analyses. If this threshold had been met, publication bias would have been evaluated using funnel plot inspection and Egger’s regression test. Because fewer than 10 studies were available for each pooled outcome in the present review, formal publication bias testing was not performed.

*Certainty of evidence*

The certainty of evidence for each pooled postoperative visual acuity outcome (UDVA, UIVA, and UNVA at 3 months) was assessed using the GRADE (Grading of Recommendations Assessment, Development and Evaluation) framework within the R version 4.5.2 (R Foundation for Statistical Computing, Vienna, Austria) using RStudio (Posit Software, PBC, Boston, MA, USA), version 2023.12.1 + 402 [24,25] analytical workflow [26,27,28]. GRADE evidence profiles were developed for each meta-analytic outcome and summarized in a structured table.

Following GRADE guidance, certainty assessment was conducted in a stepwise manner:

1. Initial rating by study design

Because all included studies contributing to the quantitative synthesis were observational/non-randomized, each outcome started at low certainty.

2. Risk of bias assessment

Certainty was evaluated for possible downgrading based on methodological limitations identified in the included studies (informed by the MMAT).

3. Inconsistency

Certainty was assessed for inconsistency across studies using the direction and magnitude of effects, overlap of confidence intervals, and statistical heterogeneity indicators (particularly I^2^ and τ^2^).

4. Indirectness

The evidence was assessed for indirectness with respect to the review question, including population, intervention (mix-and-match IOL implantation strategy), and outcome measurements (logMAR visual acuity at 3 months).

5. Imprecision

Certainty was evaluated for imprecision based on the width of pooled confidence intervals and whether the pooled estimates were sufficiently precise for interpretation.

6. Publication bias

Publication bias was considered as a GRADE domain; however, because fewer than 10 studies were available per outcome, formal statistical assessment was not feasible, and publication bias was recorded as not assessed.

### 2.5. Risk of Bias

The methodological quality and risk of bias of the included studies were evaluated using the Mixed Methods Appraisal Tool (MMAT). This instrument enables a comprehensive assessment of qualitative, quantitative, and mixed-methods study designs through a set of design-specific criteria. Each study was appraised according to the relevant MMAT category, focusing on the appropriateness of the study design, adequacy of data collection methods, rigor of outcome measurements, and coherence between research questions, methodology, and reported results. The overall methodological quality was determined based on the extent to which the MMAT criteria were satisfied, with higher compliance indicating a lower risk of bias. The appraisal process was performed independently by two reviewers (M.R. and I.K.) following prior agreement on the assessment framework and interpretation of the MMAT criteria. Any disagreements were resolved through discussion until consensus was achieved (Supplemental Table S4).

As all included studies were quantitative, non-randomized investigations, the MMAT criteria for non-randomized studies were applied consistently across studies. The first two screening questions were used to confirm that each study had a clearly defined research question and that the collected data were adequate to address it. In line with MMAT recommendations, no overall numerical quality score was calculated. Overall, all studies met at least four of the five remaining criteria.

## 3. Results

### 3.1. Study Selection and Characteristics of the Included Studies

**Figure 1:**
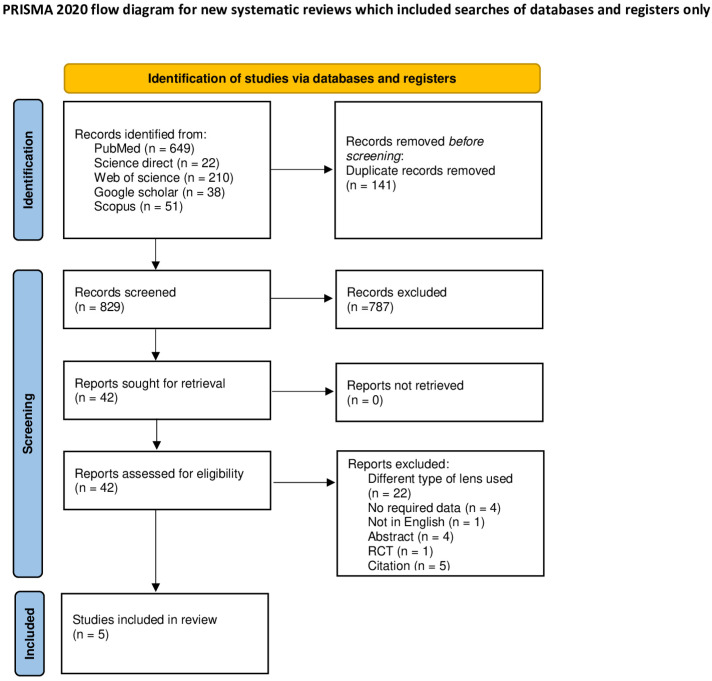
PRISMA flowchart of study inclusion.

A total of 405 articles were identified using the search strategy described above. After removing duplicates, 264 titles and abstracts were screened, of which 42 articles were selected for full-text evaluation. Full text was available for all studies. Following a full-text assessment, 5 studies met the PICOS eligibility criteria and were included in the meta-analysis [29,30,31,32,33,34]. Among the excluded articles, 22 were excluded from the final analysis due to the use of intraocular lenses of different optical designs that were outside the scope of this systematic review [17,33,35,36,37]. Furthermore, one study was excluded due to publication in the Russian language [38], one study was a randomized controlled trial [39], and four studies did not include the required data [40,41,42,43]. The full dataset analyzed for this study is presented in Supplementary Table S5. The PRISMA flowchart illustrating the study selection and inclusion process is presented in Figure 1.

Table 1 summarizes the main characteristics of the included studies. A total of six studies published between 2021 and 2025 were included, representing diverse geographical regions including Korea, Turkey, China, Greece, and the United States. Most studies were single-center investigations with predominantly prospective or retrospective non-randomized designs. The sample sizes ranged from 20 to 102 patients. The mean age of participants varied between 55.0 and 66.1 years, reflecting a typical cataract population. All studies evaluated mix-and-match implantation strategies specifically combining trifocal and extended depth of focus (EDOF) intraocular lenses to optimize binocular visual performance across different distances. Overall, the included studies demonstrated methodological heterogeneity in design and population size, while all studies focused on the same clinical strategy of combining trifocal and extended depth of focus (EDOF) intraocular lenses.

### 3.2. Meta-Analysis Results

**Figure 2:**
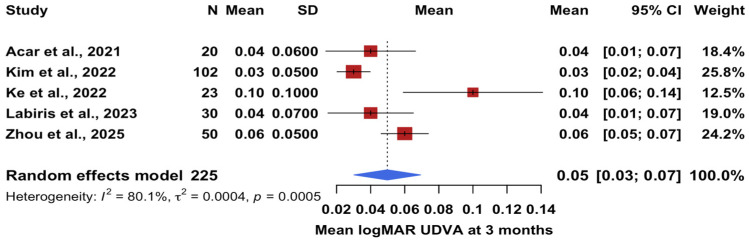

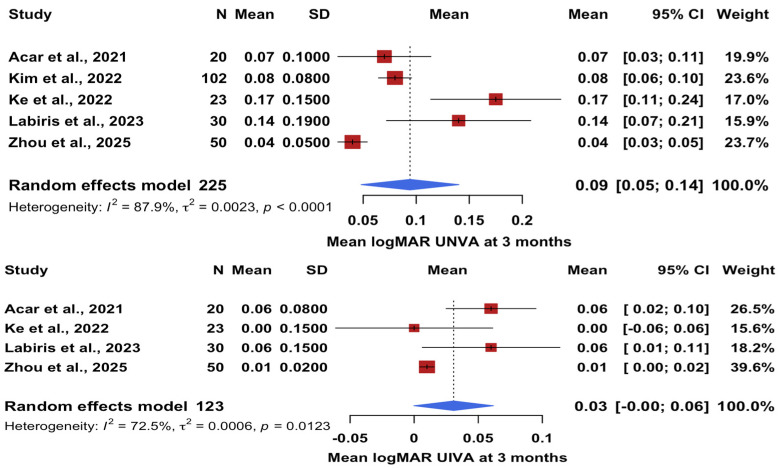
Forest Plots of Pooled Visual Acuity Outcomes at 3 Months. Studies included to the meta-analysis: Acar 2021 [29], Kim 2022 [30], Ke 2022 [31], Labiris 2023 [32], Zhou 2025 [34].

A random-effects meta-analysis was conducted, and the results are presented in Figure 2. Five studies involving a total of 225 patients were included for distance and near visual acuity outcomes, while four studies (*n* = 123) were included for intermediate visual acuity. The pooled mean logMAR UDVA at 3 months was 0.05 (95% CI: 0.03–0.07), with substantial heterogeneity (I^2^ = 80.1%, τ^2^ = 0.0004, *p* = 0.0005). The pooled mean logMAR UNVA was 0.09 (95% CI: 0.05–0.14), demonstrating high heterogeneity (I^2^ = 87.9%, τ^2^ = 0.0023, *p* < 0.0001). For intermediate vision, the pooled mean logMAR UIVA was 0.03 (95% CI: −0.00–0.06), with moderate heterogeneity (I^2^ = 72.5%, τ^2^ = 0.0006, *p* = 0.0123). Overall, the observed heterogeneity across outcomes likely reflects differences in study design, patient populations, and clinical protocols.

**Figure 3:**
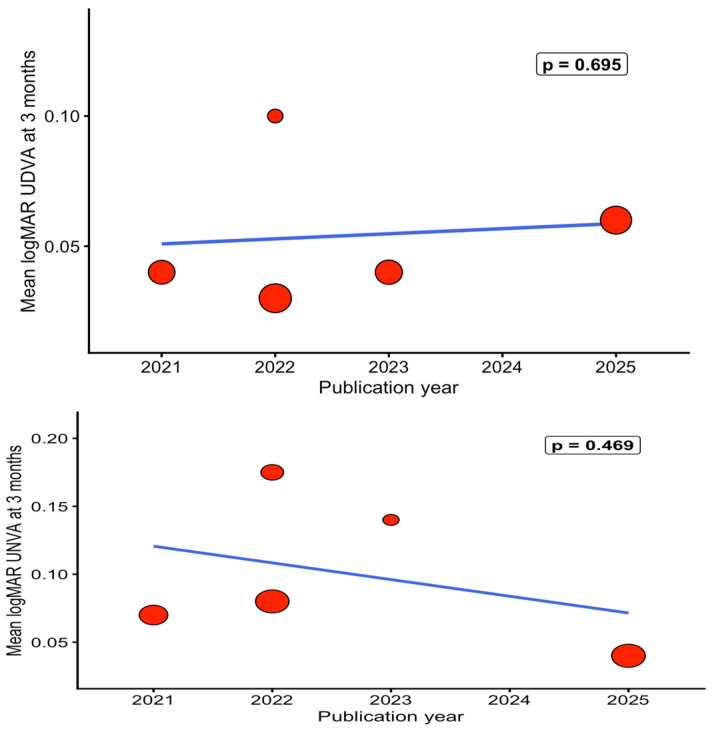

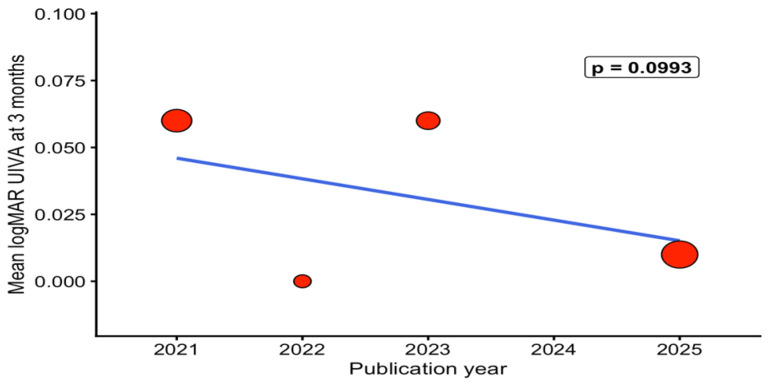
Meta-regression analysis of the pooled estimates based on the year of publication.

Meta-regression analyses were conducted using a random-effects REML model to examine whether publication year was associated with visual acuity outcomes at 3 months (Figure 3). For UDVA, a slight positive temporal trend was observed; however, this association was not statistically significant (*p* = 0.695). For UNVA, the regression analysis demonstrated a negative trend over time, which also did not reach statistical significance (*p* = 0.469). Similarly, for UIVA, a negative trend was identified, but the association remained non-significant (*p* = 0.099). Overall, these exploratory findings did not demonstrate statistically significant associations between publication year and postoperative visual acuity outcomes. Meta-regression results using mean participant age as a moderator are presented in Supplementary Figure S1; consistent with the publication-year analysis, no statistically significant associations were observed.

**Figure 4:**
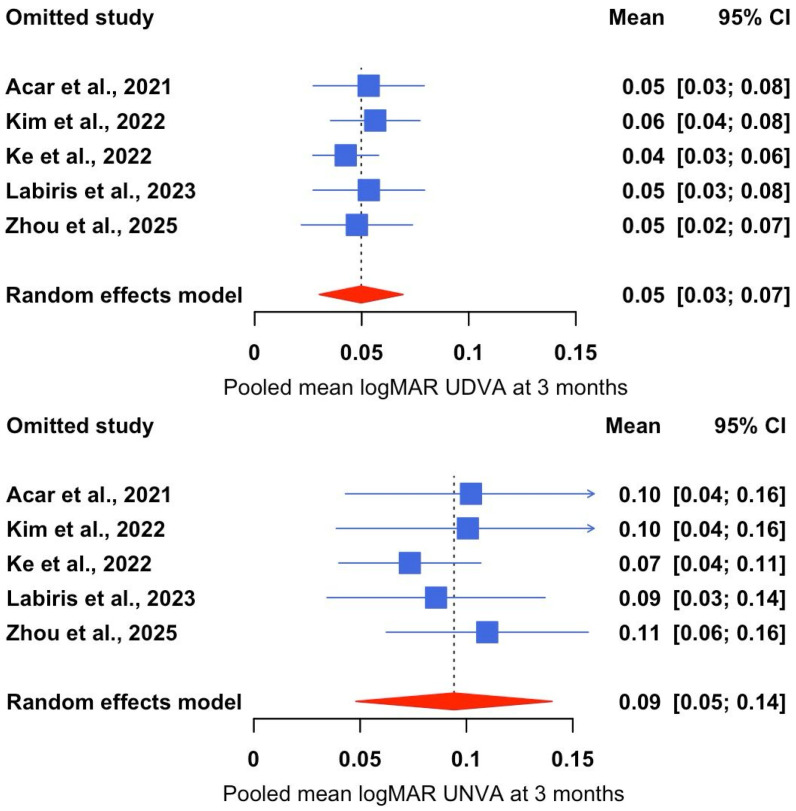

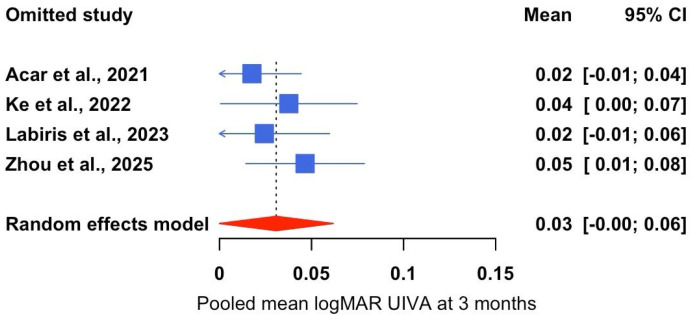
Leave-One-Out Sensitivity Analysis of Visual Acuity Outcomes. Studies included to the meta-analysis: Acar 2021 [29], Kim 2022 [30], Ke 2022 [31], Labiris 2023 [32], Zhou 2025 [34].

Leave-one-out sensitivity analyses demonstrated that the pooled estimates for UDVA, UNVA, and UIVA at 3 months were generally robust to the exclusion of individual studies (Figure 4). For UDVA, sequential removal of each study resulted in minimal changes in the pooled mean estimates, which remained within a narrow range (0.04–0.06 logMAR), indicating high stability of the findings despite substantial heterogeneity (I^2^ ≈ 80%). Similarly, for UNVA, the pooled estimates varied only slightly (0.07–0.11 logMAR) following exclusion of individual studies, suggesting that no single study disproportionately influenced the overall result, although heterogeneity remained high (I^2^ ≈ 88%). For UIVA, the pooled estimates ranged from 0.02 to 0.05 logMAR across sensitivity analyses. Although minor fluctuations in effect size and heterogeneity were observed, no single study led to a substantial shift in the overall estimate or reduction to negligible heterogeneity. Overall, these findings indicate that the meta-analysis results are stable and not driven by any single study.

The certainty of evidence assessment results using the GRADE framework is presented in Table 2. The certainty of evidence for postoperative visual acuity outcomes at 3 months was rated as very low for all three meta-analytic measures. All included studies were observational in design, which resulted in an initial low level of evidence. The overall certainty was further downgraded due to a serious risk of bias and substantial inconsistency across study results. No serious concerns were identified regarding indirectness or imprecision. Publication bias was not formally assessed.

## 4. Discussion

This systematic review aimed to answer a practical clinical question: Does mix-and-match implantation of a trifocal IOL in one eye and an EDOF IOL in the fellow eye provide balanced binocular vision at different distances in the early postoperative period? Based on five observational studies including 225 patients, the overall results at approximately three months were encouraging. Distance visual acuity was stable and clinically good. Intermediate vision showed variability across studies. Near vision was more variable, but still remained within a functional range for daily tasks. These findings support the idea behind the mix-and-match strategy. The concept is to combine two different optical designs so that each eye contributes differently to the overall visual system. The trifocal lens supports near vision, while the EDOF lens strengthens intermediate performance. Our pooled results suggest that this combination does not compromise distance vision and may offer reliable intermediate outcomes. The findings of the present systematic review are generally consistent with the broader literature on presbyopia-correcting intraocular lenses [16,18,19,44,45]. Most systematic reviews and comparative studies show that modern trifocal and EDOF platforms provide comparable distance visual acuity, while differences are more pronounced at intermediate and near ranges [16]. In particular, several reviews comparing trifocal and EDOF lenses report similar distance outcomes, stronger near performance with trifocals, and relatively smoother intermediate performance with EDOF designs [19].

Our results follow a similar pattern, although within a different implantation strategy. Distance acuity remained stable, which is in line with previous bilateral implantation studies. Intermediate acuity showed variability across studies in our analysis. This likely reflects the contribution of the EDOF component within the binocular system. Near acuity remained functional but was more variable across studies, which may explain why mix-and-match results do not always reach the same near performance levels reported in bilateral trifocal implantation.

There are both theoretical and methodological reasons that may explain these differences. From a theoretical perspective, mix-and-match implantation relies on binocular integration of two different optical designs [4]. When both eyes receive identical trifocal optics, near performance is symmetrically reinforced [22]. In contrast, when only one eye carries the stronger trifocal near add, binocular near vision depends more heavily on dominance patterns, neural adaptation, and refractive targeting [46].

Methodologically, variability in testing distances for intermediate and near vision across studies likely contributes to heterogeneity. Intermediate vision may be assessed at 60, 66, 70, or 80 cm, while near testing may vary between 33 and 40 cm. Even small shifts in testing distance can influence measured acuity, particularly when comparing different optical platforms with distinct focal distributions. Differences in study populations, IOL combinations, and follow-up timing may further contribute to inconsistencies.

Another important consideration is outcome reporting. Previous reviews have highlighted that parameters such as contrast sensitivity, dysphotopsia, and patient satisfaction are often measured using different scales or subjective grading systems [47]. This limits direct comparison across studies and may explain why some meta-analyses show conflicting conclusions regarding visual quality. In our analysis, we focused primarily on binocular uncorrected visual acuity, which provides a more standardized and comparable outcome, but does not fully capture visual experience.

Overall, our findings do not contradict the previous literature; rather, they extend it. While earlier studies have mainly compared bilateral implantation strategies, our results suggest that combining different optical designs between eyes can achieve a balanced functional outcome, particularly at intermediate distances, without compromising distance acuity. At the same time, the greater variability observed for near vision indicates that patient selection and lens pairing remain important factors in optimizing outcomes.

The results of this analysis have several practical implications for clinical decision-making in cataract surgery. Mix-and-match implantation appears to provide stable distance vision and relatively favorable intermediate performance in the early postoperative period, while maintaining functional near acuity. For patients whose daily visual tasks rely heavily on intermediate distances, such as computer work, dashboard viewing, or handheld device use, this strategy may offer a balanced alternative to bilateral implantation of a single presbyopia-correcting platform.

In clinical practice, one of the main challenges when selecting presbyopia-correcting IOLs is managing the trade-off between range of vision and optical side effects. The mix-and-match approach may help distribute optical strengths between eyes rather than concentrating all visual demands on one design. However, these findings should not be interpreted as suggesting that this strategy is universally superior. Patient selection remains essential. Visual expectations, ocular surface status, macular health, refractive targeting strategy, and tolerance for potential photic phenomena must be carefully evaluated before surgery.

Importantly, the greater variability observed in near outcomes suggests that not all patients experience the same level of near performance. This highlights the need for detailed preoperative counseling. Patients should understand that while the overall visual range may be broad, near sharpness may not fully replicate bilateral trifocal implantation in every case.

Several limitations of this review should be considered when interpreting the findings. First, all included studies were observational in design. While this reflects the current state of the available evidence on mix-and-match implantation, it may introduce potential selection bias and residual confounding. Most studies demonstrated acceptable methodological quality; however, the absence of randomized allocation and masking limits the strength of causal inference. According to the GRADE framework, the overall certainty of evidence was rated as very low, mainly due to study design considerations and observed heterogeneity rather than major methodological flaws. Therefore, the results of this study should be interpreted with caution. Second, moderate to high heterogeneity was observed, particularly for near visual acuity. This variability likely reflects differences in IOL combinations, optical platforms, surgical techniques, refractive targeting strategies, and patient characteristics. In addition, testing distances for intermediate and near vision were not fully standardized across studies. Third, the relatively small number of studies included limited the ability to formally assess publication bias. Furthermore, only studies published in English were included, which may introduce language bias. Fourth, the analysis was restricted to early postoperative outcomes at three months. Long-term stability, refractive shifts, neuroadaptation processes, and patient-reported satisfaction were not consistently evaluated. Visual acuity alone does not fully capture overall visual quality, and important parameters such as contrast sensitivity, defocus curves, and dysphotopsia severity were not uniformly reported in a way that allowed quantitative synthesis. Another important source of heterogeneity was the inconsistent reporting of ocular dominance and IOL allocation strategy. In mix-and-match implantation, the assignment of a trifocal or EDOF IOL to the dominant or non-dominant eye may influence binocular visual outcomes, particularly intermediate and near visual acuity. However, several included studies did not assess or report ocular dominance, and refractive targeting strategies, including micromonovision, were inconsistently described. Therefore, subgroup analysis according to dominant-eye allocation or micromonovision strategy was not possible.

Despite these limitations, this review has several methodological strengths. A predefined protocol was followed, and study selection and data extraction were performed systematically. The use of random-effects models allowed for between-study variability. Sensitivity analyses and influence diagnostics were conducted to evaluate the robustness of pooled estimates. In addition, meta-regression was used to explore potential moderators, including patient age and year of publication, which did not significantly influence outcomes. Together, these methodological approaches enhance the reliability of the findings within the constraints of the available evidence.

## 5. Conclusions

This systematic review and meta-analysis suggest that mix-and-match implantation of a trifocal IOL in one eye and an EDOF IOL in the fellow eye provides favorable early binocular visual acuity across distance, intermediate, and near ranges. Distance vision remained stable, while intermediate and near visual outcomes showed greater variability across studies. These findings are clinically relevant, as they support the concept of combining different optical designs to achieve a broader range of functional vision. However, all included studies were observational, heterogeneity was substantial, and the certainty of evidence was rated as very low for all outcomes. Meta-regression analyses did not demonstrate statistically significant associations between study characteristics and visual outcomes. Therefore, these findings should be interpreted cautiously and should not be considered definitive evidence of superiority over other bilateral implantation strategies. Further well-designed prospective comparative studies with standardized protocols, longer follow-up, and comprehensive assessment of visual quality and patient-reported outcomes are needed to better define the role of mix-and-match implantation in clinical practice.

## Figures and Tables

**Table 1:** Characteristics of the included studies.

Last Name, Year	Design	Country	Patients	Mean Age	Procedure	Testing Distance	Dominant/Non-Dominant Eye
Acar, 2021 [29]	Retrospective, non-randomized comparative	Turkey	20	66.1 ± 4.2	AT LARA 829 MP and AT LISA tri 839 MP	UIVA 60 cm and 80 cm; UNVA 40 cm	EDOF IOL in the dominant eye and a trifocal IOL in the non-dominant eye
Kim, 2021 [30]	Retrospective registry analysis	Korea	102	55.0 ± 5.4	AT LARA 829 MP and AT LISA tri 839 MP	UNVA 40 cm	AT LISA tri839 MP trifocal IOL was preferred for eyes with smaller preoperative corneal astigmatism, and the AT LARA 829 MP trifocal EDOF IOL was preferred for the dominant eye at the discretion of the surgeon.
Ke, 2022 [31]	Single-center, prospective case series	China	23	62.14 ± 6.3	AT LISA tri839 MP and ZXR00	UIVA 80 cm; UNVA 40 cm	No distinction between the dominant and non-dominant eyes.
Labiris, 2023 [32]	Prospective, comparative, non-randomized trial	Greece	30	63.17 ± 6.18	Clareon PanOptix and Clareon Vivity	UIVA 60 cm; UNVA 40 cm	EDOF IOL in the dominant eye and a trifocal IOL in the non-dominant eye
Zhou, 2025 [34]	A single-armed, non-interventional, ambispective case series	USA	50	57 ± 6	Clareon PanOptix and Clareon Vivity	UIVA 60 cm; UNVA 40 cm	EDOF IOL in the dominant eye and a trifocal IOL in the non-dominant eye

**Table 2:** Evaluation of the certainty of evidence using the GRADE framework.

Outcome	Study Design	Risk of Bias	Inconsistency	Indirectness	Imprecision	Publication Bias	Overall Certainty
UDVA (Far vision)	Observational	Serious	Serious	Not serious	Not serious	Not assessed	Very low
UNVA (Near vision)	Observational	Serious	Serious	Not serious	Not serious	Not assessed	Very low
UIVA (Intermediate vision)	Observational	Serious	Serious	Not serious	Not serious	Not assessed	Very low

## Data Availability

The original contributions presented in this study are included in the article and its Supplementary Materials. Further inquiries can be directed to the corresponding author (Z.U.).

## Supplementary Materials

The following supporting information can be downloaded at https://www.mdpi.com/article/10.3390/medicina62061112/s1. Supplementary Figure S1: Meta-regression of pooled estimates based on the mean age of the participants. Supplemental Table S1. PRISMA 2020 Checklist. Supplemental Table S2. Search strategy. Supplemental Table S3. Eligibility Criteria. Supplemental Table S4. Risk of bias assessment results. Supplemental Table S5. Combined data analyzed for the present study.

## Abbreviations

The following abbreviations are used in this manuscript:

IOL	Intraocular lens
EDOF	Extended depth-of-focus
UCVA	Uncorrected visual acuity
PICOS	Population, Intervention, Comparator, Outcome, and Study Design
UDVA	Uncorrected distance visual acuity
UIVA	Uncorrected intermediate visual acuity
UNVA	Uncorrected near visual acuity
GRADE	Grading of Recommendations Assessment, Development and Evaluation
MMAT	Mixed Methods Appraisal Tool
