# Supplementary material for: Visual Outcomes After Mix-and-Match Implantation of Trifocal and Extended Depth-of-Focus Intraocular Lenses: A Systematic Review and Meta-Analysis
Source: Medicina (Kaunas). 2026 Jun 8;62(6):1112. doi: 10.3390/medicina62061112 (PMC13304290; doi:10.3390/medicina62061112)
Supplement: Supplementary file 1 [file medicina-62-01112-s001.zip › Supplemental Table S2.pdf]

Search strategy of the Visual Outcomes After Mix-and-Match Implantation of Trifocal and Extended Depth-of-Focus Intraocular Lenses: A Systematic Review and Meta-Analysis.

PubMed	Web of Science	Scopus	Science Direct/or other	Google Scholar
**Criteria: Search in All Fields.**<br>**Filter: Language: English, Species: Human**<br>**No filter on the year of publication**<br>**Search date: 26 December 2025**<br><br>1. "mix and match implantation" OR "mix & match implantation" OR "mix-and-match implantation" AND ("Intraocular Lenses"[MeSH] OR "intraocular lens" OR "intraocular lenses" OR IOL OR IOLs) AND "trifocal intraocular lens" OR "trifocal IOL" OR "extended depth of focus" OR EDOF) AND ("Cataract"[MeSH] OR cataract OR "age-related cataract")	**Criteria: Search in All Fields.**<br>**Filter: Include only "Articles"**<br>**Search date: 25 December 2025**<br><br>1. "mix and match implantation" OR "mix & match implantation" OR "mix-and-match implantation"	**Criteria: Search in All Fields.**<br>**Filter: Include only "Scholarly Journals" & "Peer-Reviewed"**<br>**Search date: 25 December 2025**<br><br>1. "mix and match" AND "lens implantation" OR "mix and match" AND "intraocular lens" OR "mix & match" AND "multifocal intraocular lens" OR "mix & match" AND "lens implantation" OR "mix & match" AND "intraocular lens"	**Criteria: Search in All Fields.**<br>**Filter: Include only "Academic Journals"**<br>**Search date: 25 December 2025**<br><br>1. "mix and match implantation" OR "mix & match implantation" OR "mix-and-match implantation"	**Criteria: Search in title and full text/Search all fields.**<br>**Search date: 26 December 2025**<br><br>1. "mix and match implantation" OR "mix & match implantation" OR "mix-and-match implantation"
