# Supplementary material for: Visual Outcomes After Mix-and-Match Implantation of Trifocal and Extended Depth-of-Focus Intraocular Lenses: A Systematic Review and Meta-Analysis
Source: Medicina (Kaunas). 2026 Jun 8;62(6):1112. doi: 10.3390/medicina62061112 (PMC13304290; doi:10.3390/medicina62061112)
Supplement: Supplementary file 1 [file medicina-62-01112-s001.zip › Supplemental Table S3.pdf]

Table 2. Study eligibility criteria based on the PICOS framework

PICOS framework	Inclusion criteria	Exclusion criteria
Population	Adult patients diagnosed with age-related cataracts undergoing cataract surgery with bilateral intraocular lens implantation.	Patients with congenital, traumatic, or complicated cataracts; pediatric population; patients with ocular comorbidities potentially affecting postoperative visual outcomes (advanced glaucoma, age-related macular degeneration, diabetic retinopathy); history of previous refractive or intraocular surgery.
Intervention	Mixed implantation strategy involving contralateral implantation of multifocal intraocular lenses (MIOLs) and extended depth of focus (EDOF) intraocular lenses.	Unilateral implantation, bilateral implantation of the same IOL type, or implantation of monofocal intraocular lenses only.
Comparator	Not applicable.	Not applicable.
Outcome	Postoperative **uncorrected** distance, intermediate, and near visual acuity (UDVA, UIVA, UNVA, mean +- standard deviation values) reported in logMAR at 3 months after surgery.	Studies reporting only corrected visual acuity, optical quality metrics, patient-reported outcomes, or adverse events without uncorrected visual acuity data.
Study design	Prospective and retrospective non-randomized interventional studies and descriptive quantitative studies with outcome data.	RCTs, Reviews, Abstracts, Case Reports and Editorials. Non-English studies were also excluded.
