# Supplementary material for: Visual Outcomes After Mix-and-Match Implantation of Trifocal and Extended Depth-of-Focus Intraocular Lenses: A Systematic Review and Meta-Analysis
Source: Medicina (Kaunas). 2026 Jun 8;62(6):1112. doi: 10.3390/medicina62061112 (PMC13304290; doi:10.3390/medicina62061112)
Supplement: Supplementary file 1 [file medicina-62-01112-s001.zip › Supplemental Table S4.pdf]

Table 3. MMAT risk of bias assessment results

Study	S1	S2	C1	C2	C3	C4	C5
**Acar 2021** [29]	Yes	Yes	Yes	Yes	Yes	No	Yes
**Ke 2022** [31]	Yes	Yes	Yes	Yes	No	No	Yes
**Lee 2021** [33]	Yes	Yes	Yes	Yes	Yes	Can't tell	Yes
**Kim 2021** [30]	Yes	Yes	Yes	Yes	Can't tell	Yes	Yes
**Zhou 2025** [34]	Yes	Yes	Yes	Yes	Yes	Can't tell	Yes
**Labiris 2023** [32]	Yes	Yes	Yes	Yes	Yes	No	Yes
