# Supplementary material for: Visual Outcomes After Mix-and-Match Implantation of Trifocal and Extended Depth-of-Focus Intraocular Lenses: A Systematic Review and Meta-Analysis
Source: Medicina (Kaunas). 2026 Jun 8;62(6):1112. doi: 10.3390/medicina62061112 (PMC13304290; doi:10.3390/medicina62061112)
Supplement: Supplementary file 1 [file medicina-62-01112-s001.zip › Supplementary figure S1.pdf]

A

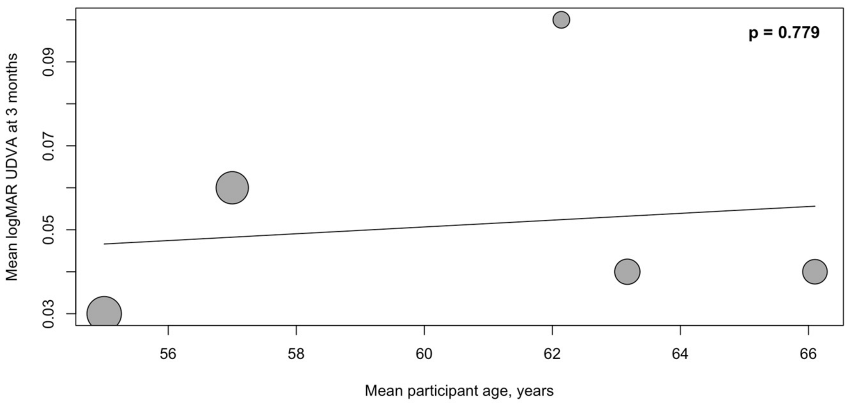

B

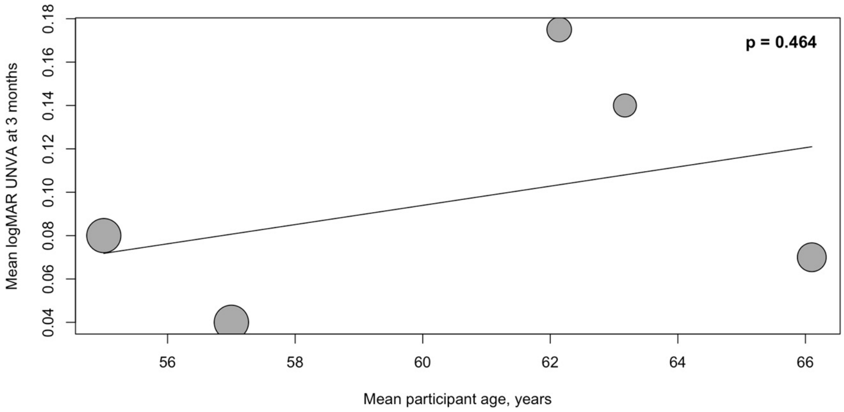

C

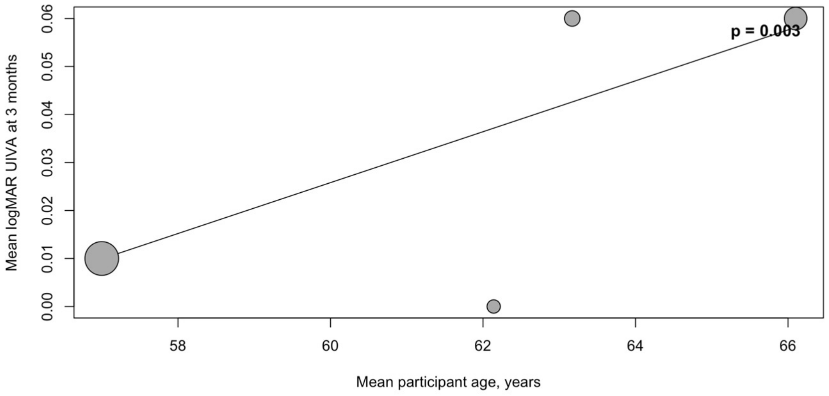

Figure S1. Meta-regression of pooled estimates based on the mean age of the participants: A) UDVA; B) UNVA; C) UIVA
